# Prurigo Pigmentosa: A Case Report With Unusual Presentation

**DOI:** 10.7759/cureus.32242

**Published:** 2022-12-06

**Authors:** Elaf R Altalhi, Muruj S Azhari, Sara S Aljuhani, Badee A Baltow, Khalid Al Hawsawi

**Affiliations:** 1 Medicine, Umm Al-Qura University, Makkah, SAU; 2 Pathology, Laboratory and Blood Bank, King Abdullah Medical City (KAMC), Makkah, SAU; 3 Dermatology, King Abdulaziz Hospital, Makkah, SAU

**Keywords:** reticulated pigmentary disorders, reticulated hyperpigmentation, keto rash, nagashima disease, prurigo pigmentosa

## Abstract

Prurigo pigmentosa (PP) is an idiopathic cutaneous inflammatory disorder. Here we report a 50-year-old healthy male of Arabic descent who presented with a six-month history of very itchy persistent skin lesions on his back. Skin examination revealed multiple brownish non-scaly excoriated papules and patches in the midline of his lower back. The differential diagnosis includes lichen planus (LP), confluent and reticulated papillomatosis (CARP), and PP. Skin biopsy revealed acanthosis, spongiosis, and dyskeratotic keratinocytes in the epidermis. The dermis showed mild perivascular lymphocytic infiltrate. Based on the previous clinicopathological findings, the patient was diagnosed with PP. He was prescribed doxycycline 100 mg once daily (OD) for two months. Two months after treatment, all lesions disappeared completely. After one year at the follow-up, he presented with a recurrence of the same skin lesions at the same site. We restarted him on doxycycline treatment.

## Introduction

Prurigo pigmentosa (PP) is an idiopathic cutaneous inflammatory disorder. It primarily affects adolescents and young adults. It is characterized clinically by a recurrent, sudden appearance of itchy, erythematous papules, macules, and/or papulovesicles on the back, neck, and chest that occur in crops. Healing of lesions occurs within weeks leaving macular reticulate hyperpigmentation. Prurigo pigmentosa is commonly seen in Japanese women; much fewer cases have been reported worldwide without predominant ethnicity [1]. The etiology of PP is not fully understood. However, there are some endogenous factors and exogenous factors that have been implicated in the pathogenesis of the disease [2]. Here we present an unusual case of PP that presented as small lesions in the midline in the sacral area in the lower back.

## Case presentation

**Figure 1:**
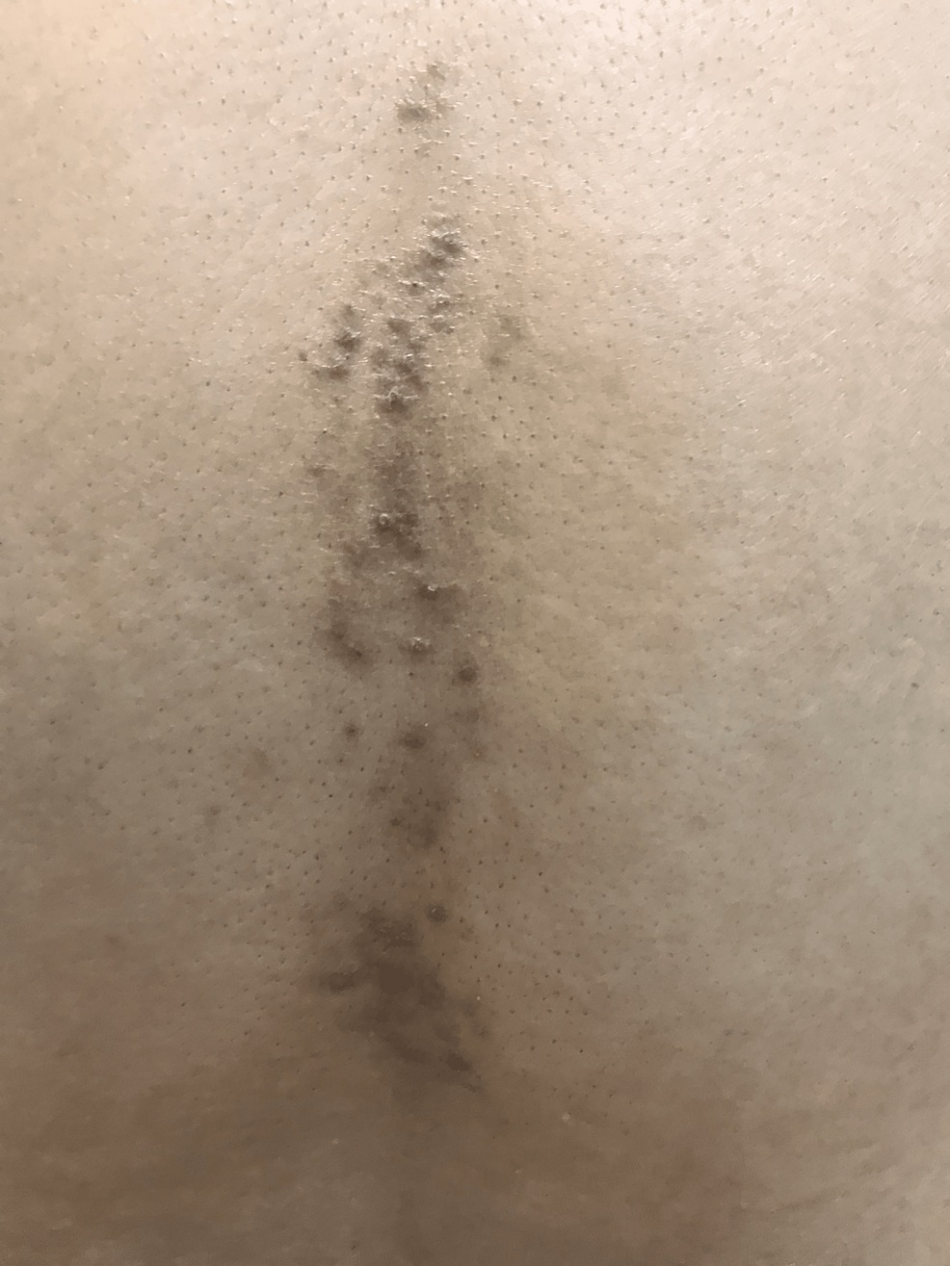
The lower back of the patient with multiple brownish non-scaly papules and hyperpigmented patches that are confined to the midline.

**Figure 2:**
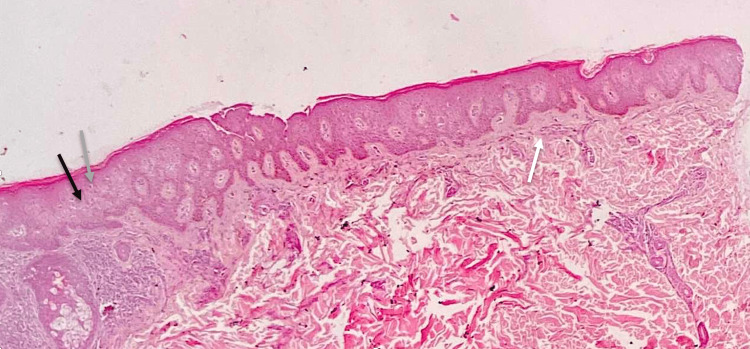
Punch skin biopsy from the lesions (hematoxylin and eosin stain; original magnification x20) shows acanthosis, mild spongiosis (black arrow), and dyskeratotic keratinocytes (gray arrow) in the epidermis. The dermis shows a very mild superficial perivascular lymphocytic infiltrate (white arrow).

**Figure 3:**
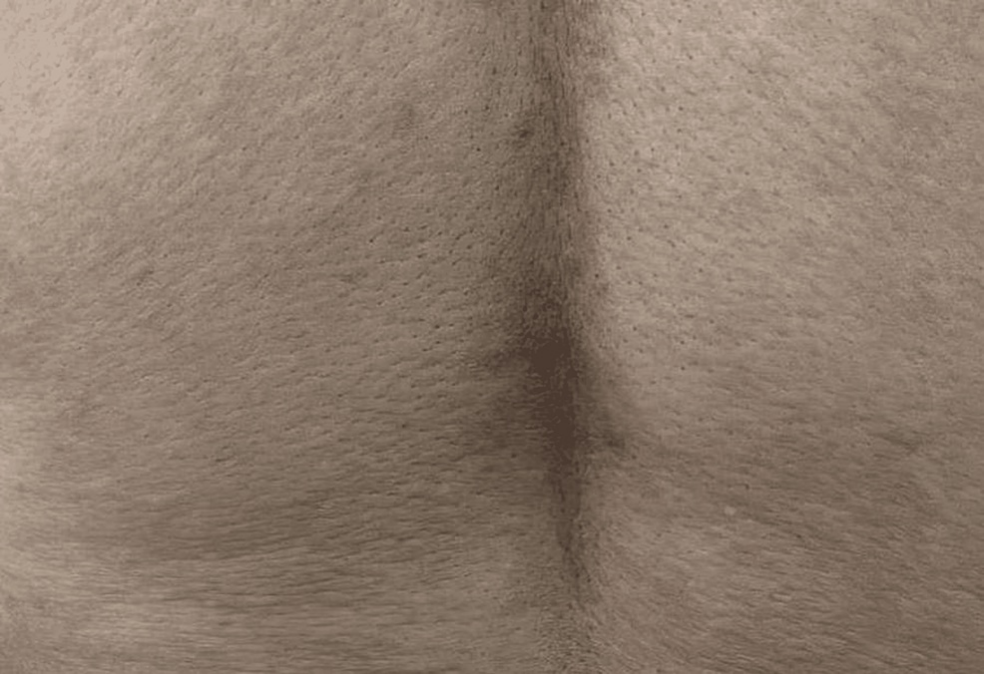
The back of the patient after doxycycline treatment showing complete healing of the lesions leaving reticulated hyperpigmented patches.

A 50-year-old male of Arabic descent presented with a six-month history of very itchy persistent skin lesions on his back. Past medical history, drug history, and review of systems were unremarkable. There is no similar case in the family. Skin examination revealed multiple brownish non-scaly excoriated papules and patches in the midline of his lower back (Figure 1). The differential diagnosis includes lichen planus (LP), confluent and reticulated papillomatosis (CARP), and PP. Skin biopsy revealed acanthosis, spongiosis, and dyskeratotic keratinocytes in the epidermis. The dermis showed mild perivascular lymphocytic infiltrate (Figure 2). Hair, nail, and mucosal examination were all normal. Based on the previous clinicopathological findings, a diagnosis of PP was made. He was prescribed doxycycline 100 mg once daily (OD) for two months. Two months after treatment, all lesions disappeared completely (Figure 3). At the one-year follow-up, he presented with a recurrence of the same skin lesions at the same site and was restarted on doxycycline treatment.

## Discussion

Prurigo pigmentosa is an idiopathic cutaneous inflammatory disorder. It is characterized by a recurrent sudden onset of pruritic and erythematous papules on the back, neck, and chest that heal in a reticulated pattern [3]. Like in our patient, PP occurs in multiple stages, with some in the early stage with excoriated papules and others in the late stage with reticulated hyperpigmented patches. It most commonly occurs in females in the third decade of life with a female-to-male ratio of 2-4:1 [4]. Our patient is a male in his fifth decade of life who presented with pruritic non-erythematous brownish non-scaly excoriated papules in the lumbosacral area which is a rare location. Moreover, they were confined to the midline which is an unusual feature.

The main differential diagnosis in our patient includes LP, CARP, and PP. However, the clinical presentations of these entities are different. Confluent and reticulated papillomatosis is characterized by non-pruritic hyperpigmented papules and plaques that are confluent in the center and reticulated at the periphery. Although our patient had midline lesions which are typical for CARP, the morphology of the lesions and history of very itchy lesions are typical for PP. Histopathologically, papillomatosis is a typical feature of CARP which was not present in our case. Although PP has been reported with ketoacidosis in poorly controlled diabetes as well as ketosis following a restrictive calorie or low carbohydrate diet, our patient had none of these. Table 1 shows the differentiations between PP and CARP. 

**Table 1:** Differentiation between confluent and reticulated papillomatosis (CARP) and prurigo pigmentosa (PP)

	CARP	PP
Etiology	Unclear. Genetic mutation, UV radiation, endocrine disorders e.g., insulin resistance, diabetes mellitus, hypothyroidism, pituitary, menstrual irregularities, aberrant reaction to *Malassezia furfur *or *Dietzia* spp., or abnormal keratinization [5].	Unknown but associated with diabetes mellitus, nutritional deficiency, fasting, dieting, bariatric surgery, anorexia nervosa, adult-onset Still disease, pregnancy, friction with clothes, and exogenous factors like nickel, chrome, and para-amino compounds [6].
Clinical features	Multiple hyperpigmented scaly macules or papules. Forming a confluent plaque centrally, and reticulations at the periphery [5]^. ^	Sudden appearance of pruritic and erythematous papules and macules on the back, neck, and chest that heal in a reticulated pattern [6].
Histopathology	Hyperkeratosis, acanthosis, and papillomatosis	3 patterns: (1) spongiotic, (2) lichenoid, (3) acanthotic
Treatment	Systemic tetracyclines especially minocycline. Systemic retinoids. Topical retinoid, antifungal, tacrolimus, salicylic acid, hydroquinone, 5-fluorouracil, and calcipotriol [5].	1^st^ line: Minocycline 50-100 mg daily; 2^nd^ line: Tetracycline, or doxycycline, macrolide antibiotics dapsone, isotretinoin, or acitretin, topical retinoids, and tacrolimus [7].

The first-line treatment of PP is oral minocycline. However, our patient responded well to doxycycline. Prurigo pigmentosa does not respond to topical or systemic corticosteroids or antihistamines.

## Conclusions

Prurigo pigmentosa is an idiopathic cutaneous inflammatory disorder. It is clinically characterized by recurrent sudden onset pruritic and erythematous papules that occur in crops and heal in a reticulated pattern. Sometimes PP and CARP look similar, as in our case, however, these clinical entities can be distinguished by their typical clinical and histopathological features. Our patient presented with very itchy brownish excoriated papules with brownish patches that were confined to the midline on his lower back. The purpose of this case report is to raise awareness of this condition. We recommend additional research with a higher level of evidence to investigate and assess this condition. 
